# Pathophysiology of ocular toxoplasmosis: Facts and open questions

**DOI:** 10.1371/journal.pntd.0008905

**Published:** 2020-12-31

**Authors:** Valentin Greigert, Faiza Bittich-Fahmi, Alexander W. Pfaff

**Affiliations:** 1 Institut de Parasitologie et Pathologie Tropicale, UR 7292, Fédération de Médecine Translationnelle, Université de Strasbourg, Strasbourg, France; 2 Service de Parasitologie et Mycologie Médicale, Hôpitaux Universitaires de Strasbourg, Strasbourg, France; Hitit University, Faculty of Medicine, TURKEY

## Abstract

Infections with the protozoan parasite *Toxoplasma gondii* are frequent, but one of its main consequences, ocular toxoplasmosis (OT), remains poorly understood. While its clinical description has recently attracted more attention and publications, the underlying pathophysiological mechanisms are only sparsely elucidated, which is partly due to the inherent difficulties to establish relevant animal models. Furthermore, the particularities of the ocular environment explain why the abundant knowledge on systemic toxoplasmosis cannot be just transferred to the ocular situation. However, studies undertaken in mouse models have revealed a central role of interferon gamma (IFNγ) and, more surprisingly, interleukin 17 (IL17), in ocular pathology and parasite control. These studies also show the importance of the genetic background of the infective *Toxoplasma* strain. Indeed, infections due to exotic strains show a completely different pathophysiology, which translates in a different clinical outcome. These elements should lead to more individualized therapy. Furthermore, the recent advance in understanding the immune response during OT paved the way to new research leads, involving immune pathways poorly studied in this particular setting, such as type I and type III interferons. In any case, deeper knowledge of the mechanisms of this pathology is needed to establish new, more targeted treatment schemes.

## Introduction

*Toxoplasma gondii* is classically described as one of the most successful parasites in the world, as more than one-third of the global population are estimated to harbor *T*. *gondii*, albeit with great regional disparities, prevalence ranging from 10% to 80% [1]. The ocular presentation of this infection, ocular toxoplasmosis (OT), has long been exclusively attributed to congenital infection and neglected as a common health problem. Recent studies show that most of OT cases are indeed due to infection after birth [2] and that it can be considered as the first infectious cause of posterior uveitis worldwide, being responsible for 30% to 50% of all posterior uveitis in immunocompetent subjects [3].

OT typically presents as retinochoroiditis, sometimes associated with anterior uveitis or vasculitis [4]. It is often asymptomatic, especially when lesions are located at the outer edge of the retina, but can cause blurred vision and floaters [5]. In case of extensive lesions or particular location, such as macular or papillary involvement, the visual prognosis of the infected eye can be severe. A major problem is that even ancient lesions can reactivate at any time, considerably increasing the threat to the patient’s vision, for lifetime. The diagnosis of this infection is sometimes complicated, especially in a tropical setting, requiring clinical and biological assessments to identify the toxoplasmic origin of retinochoroiditis [6]. As to treatment, the few protocols available control parasite proliferation at the acute stage of the infection or during recurrences, but cannot eliminate the latent parasite stage, nor diminish the incidence of recurrences, especially when the infection is discovered at a late stage [7,8]. Furthermore, several of these treatments are not devoid of potentially severe side effects, such as the widely used association of pyrimethamin and sulfadiazine.

Despite this tremendous epidemiological and clinical importance, the pathophysiology of this infection remains poorly studied. Indeed, the complexity of the infectious process makes experimental studies specific to OT difficult. However, a few research groups have established models of specific aspects and have thus succeeded in exploring the pathophysiology of this infection. The present article aims at reviewing current knowledge regarding eye immunology and OT pathophysiology, but also to point out the numerous open questions, to better apprehend the specific complexity of this infection, as well as to stimulate new experimental approaches to understand its underlying mechanisms.

## Methodology

Reference was made to previous work obtained from meeting contributions and PubMed search on the subjects of eye immunology and physiopathology of ocular OT. Key features of OT pathophysiology, or of importance for apprehending it, were considered and are treated in the following order: (1) eye invasion and infection by *T*. *gondii*; (2) local immune response to *T*. *gondii* infection; and (3) specificities of South American OT. Figures were made using Inkscape and TheGimp softwares.

## The journey of a parasite

### *Toxoplasma gondii* infection and dissemination

OT is one of the consequences of systemic infection by *T*. *gondii*, both in utero, and, more often, after birth [2]. When the infection is acquired through the ingestion of food or water contaminated with *T*. *gondii*, sporozoites or bradyzoites are freed from oocysts or tissue cysts, respectively, following the combined action of bile acids, trypsins, pH, and other components present in the digestive tract [9]. Once freed, parasites invade enterothelial cells, forming a parasitophorous vacuole via the mobile junction mechanism [10]. They then transform into rapidly proliferating tachyzoites, lysing their host cells and reaching the blood stream. This phenomenon is responsible for an inflammatory response and the recruitment of polymorphonuclear cells, monocytes, and dendritic cells [11]. Parasites are also able to cross the intestinal barrier via a paracellular path [12]. When the infection occurs in utero, subsequently to a primo-infection of the mother, tachyzoites directly infect fetus-derived tissues, beginning with the syncytiotrophoblast [13]. However, there are few studies about this particular route of infection in humans.

The most commonly accepted hypothesis regarding parasite dissemination within its host is the one of the “Trojan horse.” It states that parasites take advantage of immune cell mobility, particularly dendritic cells (DCs), by invading them. Even more surprisingly, infected cells exhibit a modified phenotype, being more mobile under the influence of parasite-derived proteins, such as GRA5 [14]. This mechanism would allow parasites to be disseminated along lymphatic vessels. Moreover, parasites can disseminate along the blood stream, mostly inside cells, primarily monocytes [15,16]. As with DCs, *T*. *gondii* seems to have the ability of modifying the phenotype of infected monocytes and macrophages, enhancing their mobility, marginalization and extravasation [17–19]. Following dissemination, *T*. *gondii* is capable of invading tissues, including brain, heart, eyes and, muscles [20].

### The invasion of the eye

**Fig 1:**
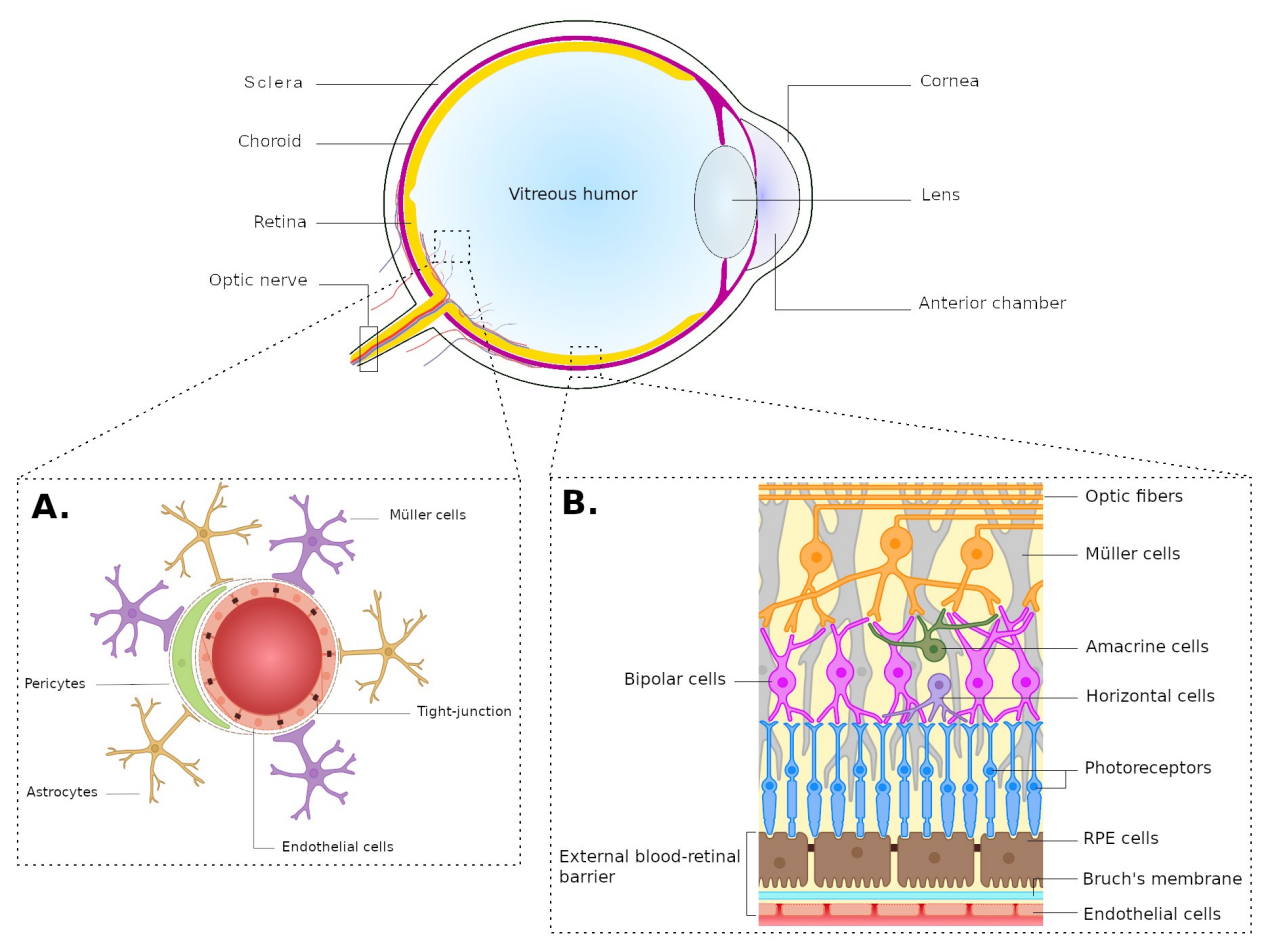
The eye and the BRBs. (A) The internal BRB isolates ocular tissues from the blood stream via tightly sealed endothelial cells, surrounded by pericytes, and Müller cell and astrocyte extensions. (B) The retina displays a stratified organization reflecting the localization of the different cell types fulfilling specific roles. The choroidal vascular system is separated from photoreceptor cells by tightly connected RPE cells, which adhere with their vascular pole to the basal Bruch membrane, forming the external BRB. BRB, blood-retinal barrier; RPE, retinal pigmented epithelial.

The eye presents a peculiar structure, composed of 3 layers (Fig 1). The outer one is known as the “fibrous tunic,” composed of the cornea and sclera, consisting mainly of collagen, protecting the eye from mechanic aggression and maintaining its shape. The middle layer is the “uvea,” which is mainly composed of vascular structures, such as the choroid and the ciliary body, but also the iris. Finally, the innermost layer is classically described as the nervous layer which is, in fact, formed by the “retina,” the sensory tissue of the eye. The eye is filled with transparent tissues and liquids, allowing the light to be properly focused onto the retina in order to form a clear image, such as the lens, the aqueous humor, located between the cornea and the lens, and the vitreous body filling the globe behind the lens [21].

Ocular tissues are supplied through 2 vascular systems, both deriving from the ophthalmic artery, the first branch of the internal carotid artery. The retinal vascular system enters the eye along the optic nerve and is made up of small blood vessels directly supplying the inner layers of the retina. The blood stream is isolated from ocular tissues via tightly sealed endothelial cells, surrounded by pericytes, and Müller cell and astrocyte extensions, forming the internal blood-retinal barrier (iBRB) (Fig 1) [22]. In primates, this barrier is only fully competent about 10 days after birth [23]. On the other hand, the choroidal vascular system enters without passing along the optic nerve. In the choroid, it forms a plexus composed of fenestrated capillaries, in charge of supplying the external layers of the retina, notably photoreceptor cells. These capillaries are separated from photoreceptor cells by tightly connected, polarized retinal pigmented epithelial (RPE) cells, which adhere with their vascular pole to the basal Bruch membrane, forming the external BRB (eBRB) (Fig 1). These RPE cells fulfill several functions, such as vitamin A metabolism, intraretinal homeostasis control, and immunoregulation. They are also responsible for transporting nutrients and metabolites across the eBRB [24]. Therefore, while this barrier also isolates the retina from the peripheral blood circulation, it is more permissive than the iBRB [22]. The principal mechanism used for the invasion of eye tissues by *T*. *gondii* has not been evaluated. In vitro studies show the ability of the parasite to cross RPE cells both in its free form, by using intercellular adhesion molecule-1 (ICAM-1)-mediated adherence [25], and within DCs, using ICAM-1, vascular cell adhesion molecule-1 (VCAM-1), and the activated leukocyte cell adhesion molecule (ALCAM) [26]. However, a recent study showed that parasites were more present in the inner layers of the retina, suggesting that the iBRB might be the preferred route for invasion over the eBRB [27], but could also be the consequence of parasite mobility inside the retina.

Indeed, the retina is a complex tissue, with a remarkable stratified organization, which reflects the localization of the different cell types fulfilling specific roles (Fig 1). Roughly, there are 5 types of neuronal cells in the retina, working together to transform light into a neurological signal for the brain: photoreceptor cells, horizontal cells, bipolar cells, amacrine cells, and ganglion cells. Axons of these latter cells form the optical nerve. In addition to neuronal cells, 2 types of glial cells are present within the retina. Müller cells are the main glial cells of this tissue, spreading across all the layers, acting mechanically to support the architecture of the retina. Additionally, these cells are also important in maintaining retinal homeostasis and are a component of the iBRB by sending extensions of their body around intraretinal blood vessels [28] (Fig 1). Astrocytes are also a component of this barrier and exert multiple functions ranging from homeostasis control to immune responses. In particular, astrocytes are responsible for controlling extracellular glutamate level, nitric oxide (NO) production, and cytokine expression [29]. Finally, retinal microglial cells are not glial cells but immune cells, deriving from the yolk sac, like cerebral microglial cells [30]. Retinal microglial cells derive from 2 different types of cells, the first ones expressing specific markers of macrophages (CD45^+^, Mac-1^+^, CD11b^+^, F4/80^+^, CD68^+^), the second ones with a CD11b^low^/CD45^low^ phenotype [31]. These cells seem to be homogeneously distributed in the retina [28]. They play an important role in the immune response against infections, immune regulation, and repair of damaged tissues. Their functions seem to be close to those of classical macrophages. However, they appear not to be able of expressing the class II major histocompatibility complex and to have limited abilities for antigen presentation [32].

Once in the retina, the parasite invades resident cells, but studies to identify a privileged cell type for infection gave divergent results [27,33]. While, in an in vitro model, *T*. *gondii* seems capable of crossing several layers of the retina before preferentially invade a glial cell, murine models have shown that the parasite infects both glial and neuronal cells, without preference [27,33]. Thus, the identification of cell types as privileged hosts of *T*. *gondii* could explain the discovery of parasites located far from their point of entry into the retina.

### Persistence and recurrences

Following a phase of active proliferation within the retina, clinically marked by a developing lesion, the increased pressure from the host immune system finally controls the proliferation but does not eliminate the parasite. *T*. *gondii* has the ability to persist in the invaded tissues by conversion of tachyzoites into slowly proliferating forms called bradyzoites, which are organized in tissue cysts [20]. Some parasite-derived proteins have been identified as playing a role in this transformation, such as ROP17, ROP35, and ROP38 [34]. No study has yet described invasion and cyst formation specifically in the eye, but it is probable that the mechanisms are similar as in other tissues. This phenomenon consists in the transformation of a parasitophorous vacuole into a cyst by an extensive modification of the vacuole membrane, involving the addition of several parasite proteins and glycosylation [35–38]. Furthermore, cyst-containing cells exhibit strongly modified microtubules and intermediate filaments networks [39].

Recurrences are an important feature of OT. This term refers to the fact that new foci of active retinochoroiditis develop, usually at the immediate vicinity of scars of ancient lesions. Thus, with every recurrence, the probability of visual impairment consecutive to toxoplasmosis increases [3]. It is remarkable that recurrences also readily occur in immunocompetent subjects. The pathophysiological basis of this phenomenon is poorly understood. It may be the consequence of the active liberation of tachyzoites from resident cysts [5]. Other studies suggested the rupture of senescent cysts, traumas, hormonal fluctuations, decrease in humoral or cellular immunity, pregnancy, or even eye surgery [40–42]. Clinical features of these recurrences might give us clues about the mechanisms underlying their development. Indeed, severe, highly progressive and extended lesions has been predominantly observed in elderly, pregnant, or immunodeficient patients [42–45]. Higher intraocular anti-*T*. *gondii* titers have also been correlated with limited risk for developing recurrences [46]. Together, these elements indicate that the quality of the immune response against *T*. *gondii* is critical for the development and the progression of OT recurrences. However, the mechanisms, which actually trigger the recurrence, remain unknown, and the difficulties in developing a model for studying such a sporadic phenomenon restrict the possibility for improving our understanding on the matter.

## The immune response to OT

### Immune privilege and immune response to *Toxoplasma gondii*

Like other sensitive, nonregenerative organs, such as brain and placenta, the eye is classically described as an “immune privileged organ.” This condition is defined by the limitation of local inflammation and immune cell activation, preventing irreversible tissue damage. Several underlying mechanisms are involved in this phenomenon and are responsible for a complete modification of the immune response to *T*. *gondii* infection.

As described earlier, the retina is well isolated from blood circulation via BRBs. These BRBs mechanically forbid circulatory immune cells, antibodies, and antigens to pass from one compartment to the other. Furthermore, even though choroid may function as a part of the lymphatic drainage of the eye [47,48], there is no proper lymphatic vasculature, liquids being directly drained into the venous circulation through the trabecular meshwork [49]. This feature greatly diminishes the capacity to present eye-derived antigens to T cells.

**Fig 2:**
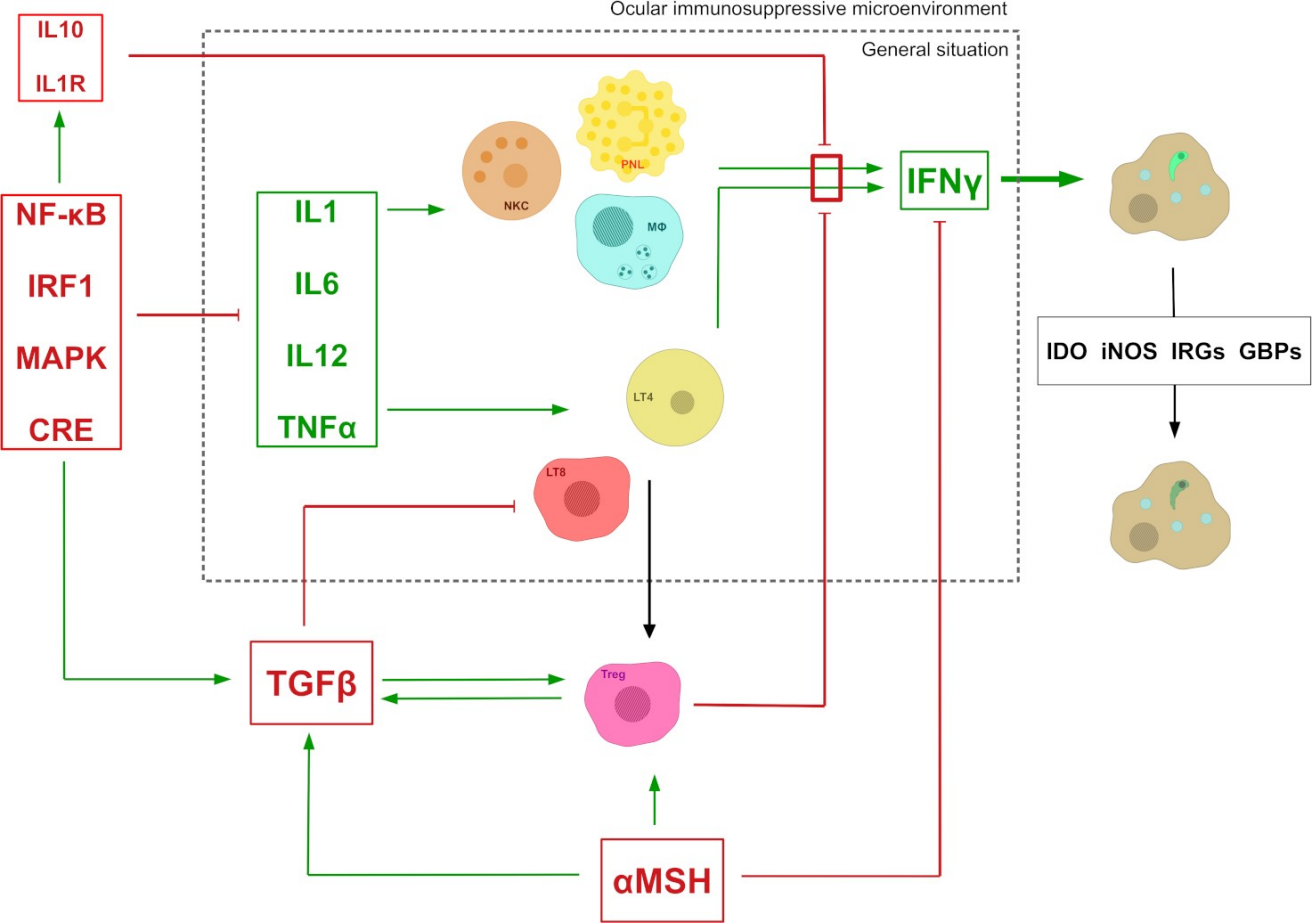
The ocular immunosuppressive microenvironment widely impairs the normal immune response to *T*. *gondii* infection. In the general situation, the lysis of the parasitophorous vacuole and, ultimately, the parasite, relies on the expression of IFNγ by multiple cell types stimulated with various T_h_1 cytokines. In the eye, this mechanism is impaired by the presence of inhibitory molecules locally expressed by retinal cells, including RPE cells. Green arrows with pointy heads mean “activates/stimulates.” Red arrows with flat heads mean “inhibits.” αMSH, α-melanocyte-stimulating hormone; CRE, cAMP response element; GBP, interferon-induced guanylate-binding protein; IDO, indoleamine 2,3-dioxygenase; IL, interleukin; IFNγ, interferon γ; iNOS, nitric oxide synthases; IRF1, interferon regulatory factor 1; IRG, immunity-related guanosine triphosphatases; LT4, CD4+ T cells; LT8, CD8+ T cells; MΦ, macrophage; MAPK, mitogen-activated protein kinase; NF-κB, nuclear factor-kappa B; NKC, natural killer cells; PNL, polymorphonuclear leukocyte; TGFβ: transforming growth factor β; TNFα, tumor necrosis factor α; Treg, regulatory T cells.

In nonimmune privileged organs, *T*. *gondii* proliferation control primarily relies on the expression of interferon gamma (IFNγ), which induces indoleamine 2,3- dioxygenase (IDO), inducible nitric oxide synthase (iNOS), effector proteins immunity-related GTPases (IRGs), and guanylate-binding proteins (GBPs), leading to inhibition of parasite growth, NO-mediated cytotoxicity, and destruction of the parasitophorous vacuole (Fig 2) [50]. Many cells are involved in the expression of IFNγ in response to *T*. *gondii* infection, such as DCs, macrophages, CD8^+^ and CD4^+^ lympocytes, granulocytes, and NK cells. In particular, CD4^+^ T_h_1 cells, activated through interleukin 12 (IL12) stimulation from antigen-presenting cells (APCs), induce a robust CD8^+^ T cell effector immunity, these cells being an important source of IFNγ and also exhibiting cytotoxic activity against infected targets [51,52]. These principles of systemic immunity to *T*. *gondii* infection have been extensively detailed in previous reviews [53,54]. In contrast, when looking at ocular infection, the posterior eye pole presents an immunosuppressive microenvironment, characterized by the expression of suppressive cytokines (Fig 2). A prominent example for such molecules is Transforming Growth Factor-β2 (TGFβ2), which regulates the differentiation, proliferation, and survival of lymphocytes [55]. In the local environment, TGFβ2 is activated and stabilized by thrombospondin-1 (TSP-1), which is constitutionally expressed in RPE cells [56]. The α-melanocyte-stimulating hormone (α-MSH) is also found in this environment and exerts its immunoregulatory functions not only by inducing the expression of TGFβ2, but also by inhibiting the expression of IFNγ and toll-like receptor 4 (TLR4), which is involved in detecting *T*. *gondii* glycosylphosphatidylinositol (GPI) during infection [53]. The vasoactive intestinal peptide (VIP) acts as a potent immunosuppressive molecule by inducing several transcription factors, such as nuclear factor kappa-light-chain-enhancer of activated B cells (NF-κΒ), interferon regulatory factor-1 (IRF-1), mitogen-activated protein kinase (MAPK), and cAMP response element (CRE). By these activations, VIP down-regulates the expression of several inflammatory molecules such as tumor necrosis factor-α (TNFα), IL1, IL6, IL12, and IFNγ, while up-regulating the expression of immunomodulatory molecules such as IL10, IL1R, and TGFβ [57]. Retinoic acid (vitamin A), detected not only in RPE cells but also in all retina layers, has been shown to have immunomodulatory functions, similar to those of TGFβ [58]. Finally, the calcitonin gene-related peptide (CGRP), which is expressed by retinal neuronal cells, has an inhibitory effect on macrophages, limiting the NO production by these cells [59].

Cellular immune mechanisms are also involved in this immunosuppressive microenvironment. α-Melanocyte-stimulating hormone (α-MSH) is able to induce the differentiation of T cells into regulatory T cells (Fig 2) [60]. Macrophages present in the eye are able to process antigens of ocular origin and to present it to T cells in the spleen marginal zone, also inducing their differentiation into regulatory T (Treg) cells [61]. Other mechanisms involve microglial cells expressing both ligands and receptors of the CD200/CD200R pathway, preventing activation of blood-borne myeloid cells [62,63]. RPE cells are equally able to induce the differentiation of T cells into regulatory T cells, along with expressing TGFβ, through the expression of prostaglandin E2 and cytotoxic T-lymphocyte-associated protein 2 (CTLA-2) [64]. Furthermore, they express ligands for programmed cell death pathways, such as FAS ligand (FasL) and PD-L1, thus effectively eliminating activated invading immune cells, especially activated T cells [64,65]. PD-L1 is also expressed by retinal neurons in naive mice, suggesting a role of these cells and the PD-1/PD-L1 pathway in maintaining the retinal immunosuppressive microenvironment [66]. Finally, the TNF-related apoptosis-inducing ligand (TRAIL), which is also expressed in the retina, particularly at the eBRB, is equally able to induce cell death.

Thus, multiple systems ensure the particular immunosuppressive microenvironment in the eye posterior pole and provide a suitable niche for *T*. *gondii* persistence and development, which makes it difficult to generalize results of studies on other, nonocular models, to OT pathophysiology.

### Innate and adaptive responses to the ocular toxoplasmosis

In vitro studies indicated that Müller cells, when infected with *T*. *gondii*, are able to express a large panel of immune mediators, such as IL4, IL6, CCL2, CXCL2, and CXCL-8 [67]. However, classical inflammatory cytokines, known to control *Toxoplasma* proliferation, such as IL12 or IFNγ, are not expressed by these cells [67].

Murine models partially confirmed these results, showing the expression of not only IL6 but also TGFβ and β2-microglobulin, in the eye of experimentally infected animals [68]. In the same study, IL6 knock-out mice exhibited a highly susceptible phenotype, with severe retinal inflammation and high parasite burden, suggesting an important role for this particular cytokine in protecting the retina against toxoplasmic infection [68]. Another study showed local expression of IFNγ and TNFα by invading lymphocytes, as well as macrophages, during toxoplasmic uveitis [69]. Other cytokines and chemokines seem to have important roles in protecting the eye against toxoplasmic infection, such as CXCL10, IFNγ, and TNFα, as demonstrated by higher parasite burdens in the corresponding knock-out or neutralization models [70,71]. Whereas these studies looked at the response to an acute primo-infection, few groups specifically studied the immune response in case of recurrences. In a work using a murine model mimicking OT recurrences conducted in our laboratory, the important retinal inflammation and high parasite burden in susceptible C57BL/6 mice correlated with strongly expressed inflammatory and T_h_1 cytokines, such as IL6 and IFNγ [72]. In contrast, the resistant Swiss-Webster mice primarily expressed T_h_2 cytokines, such as IL31 and a rapid, strong antibody production. Neutralizing cytokines injected concomitantly into the eye allowed us to dissect specifically the ocular immune response. While intraocular neutralization of IFNγ resulted without surprise in higher local parasite loads, locally administered anti-IL6 antibodies reversed the susceptible phenotype of C57BL/6 mice, both in terms of pathology and parasite control [72]. This is in contrast to the abovementioned protective effect of IL6 [68] and could therefore reflect the special immunologic environment within the immune privileged eye, which cannot be addressed using knock-out mice. Indeed, retinal MHC class II and PD-L1 expression are involved in suppressing T-cell activation following retinal toxoplasmic infection, therefore protecting the retina from CD4 T-cell-mediated immune damage [73]. While this study allowed us to look at the control of parasites during artificial rechallenge, no model has yet been established to actually observe and quantify natural recurrences.

Finally, numerous data from human samples provide clues about the immune response during OT in humans. Retrospective surveys analyzed aqueous humors of OT patients and retrieved high levels of T_h_1 and inflammatory cytokines such as IL2, IFNγ, IL6, IL17, and Monocyte Chemotactic Protein-1 (MCP-1), as well as of the T_h_1 negative control cytokine IL10 [3,74,75]. Congruently, T_h_2 cytokines, such as IL13, were poorly expressed in these patients.

### The particular role of IL17 in ocular immunity

The detection of IL17, alongside the more canonical T_h_1 cytokines, drew the attention of our group to the role of T_h_17 lymphocytes in the pathophysiology of OT. The differentiation of T_h_17 lymphocytes is stimulated by TGFβ, IL1, and IL6. They exert a pro-inflammatory effect through the secretion of cytokines and are involved in autoimmune diseases (like psoriasis, psoriatic arthritis, and rheumatoid arthritis), chronic inflammation, and protective immunity against extracellular bacteria and fungi [76]. However, the functions of T_h_17 lymphocytes are not fully understood. IL17 is the major cytokine of the T_h_17 lymphocyte subpopulation. IL17 family contains 6 members, named consecutively IL17A to IL17F and 5 identified IL17 receptors (IL17RA-E) [77]. IL17E is quite apart by its weak homology with the other family members and its T_h_2-like action and is now termed IL25. IL17 receptors are present on many cell types including immune cells. Their stimulation induces the expression of various cytokines and chemokines, leading most prominently to recruitment and maturation of neutrophils. IL17 also acts on nonimmune cells such as fibroblasts and epithelial cells, among which RPE cells, and induces the production of Granulocyte Macrophage Colony Stimulating Factor (GM-CSF) and prostaglandin E2, thereby increasing the maturation of granulocytes and the inflammatory process [78].

The first indication of the central role of IL17 came from studies on human OT patients who showed strong ocular IL17A expression, in striking contrast to viral and other uveitis cases [75]. This might suggest the existence of autoimmune processes, resulting from tissue damage. Immunofluorescence staining localized this IL17A expression mainly to Müller cells, which is rather surprising, IL17A being commonly associated with T cells or natural killer (NK) cells. In fact, whereas other studies confirmed our finding of IL17 expression in the eye [79], no evidence of infiltrating bona fide T_h_17 cells (CD4^+^ IL17^+^) has yet been described. However, such T_h_17 cells have been described in the peripheral blood of OT patients [80]. Murine models have allowed the identification of IL17 as a highly expressed cytokine in case of OT, whether during acute primo-infection through intravitreal injection or using protocols experimentally mimicking OT recurrences [72,81,82]. High expression of IL17 was correlated with the development of severe retinal lesions. The IL17 concentration in aqueous humor was higher in acutely infected mice than in animals which had already been previously challenged with the parasite, while the Treg marker FoxP3 as well as T_h_1 markers and cytokines were suppressed [82]. Using neutralization experiments, we confirmed that *T*. *gondii* induced IL17 increase ocular pathology, probably again by inhibiting the Treg inducer FoxP3, and down-regulates the protective, antiparasitic IFNγ response [74]. These results show that IL17 is indeed at the center of the immune interaction in the eye during OT, influencing both pathology and parasite control.

**Fig 3:**
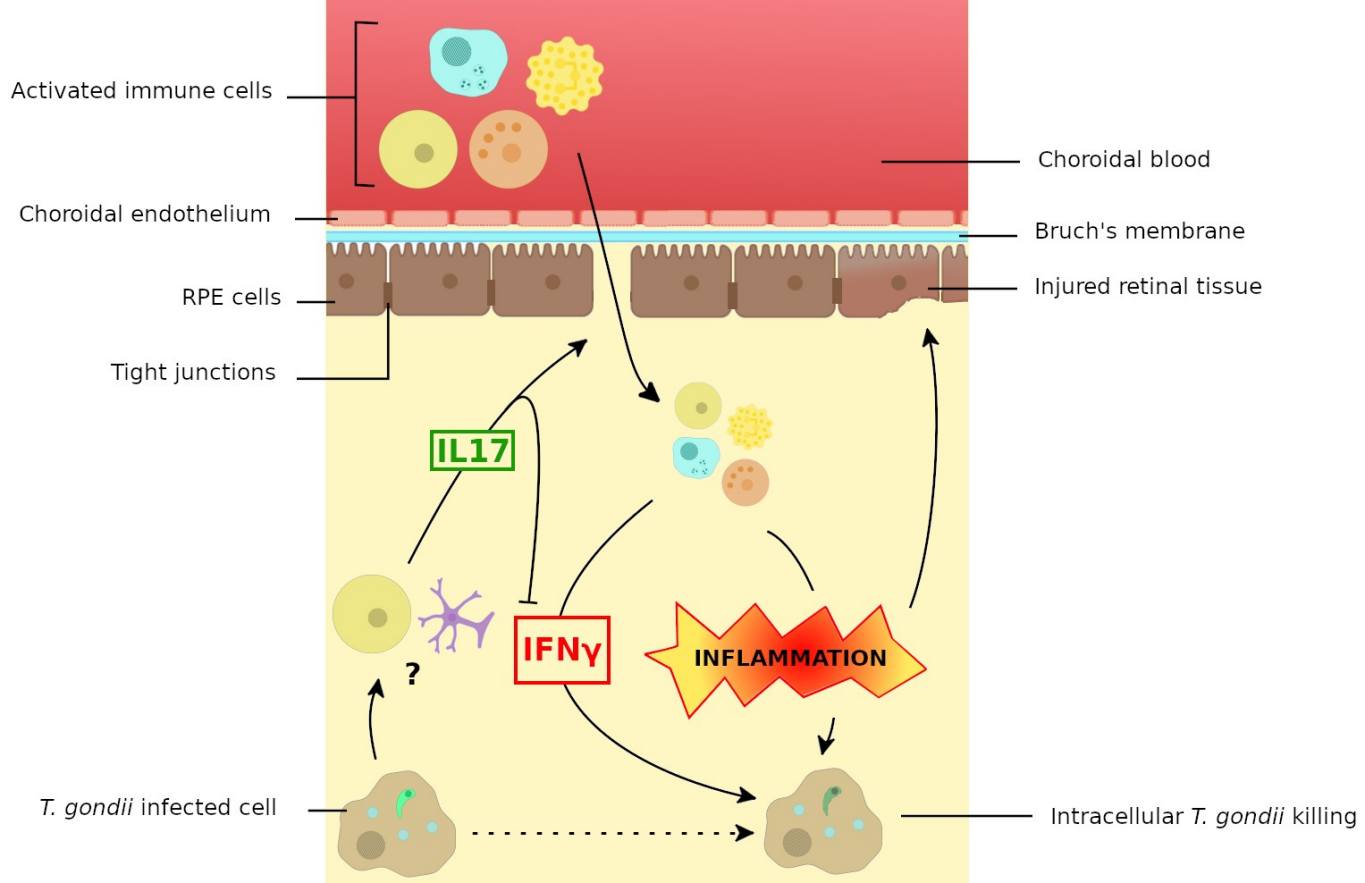
Proposed model of the immune response to the retinal infection with *Toxoplasma gondii*. Evidence suggests that the infection of retinal cells with *T*. *gondii* results in the expression of IL17 by resident cells (Müller cells, but maybe also T_h_17 T cells), which might be responsible for the recruitment into the retina of activated immune cells, facilitated by the increased permeability of the external blood-retinal barrier. These immune cells would be responsible for subsequent retinal lesions, probably also by suppression of Treg cells (not shown). At the same time, IL17 expression negatively interferes with IFNγ production, thereby diminishing the protective antiparasitic response. IFNγ, interferon gamma; IL17, interleukin 17; Treg, regulatory T cells.

An interesting hypothesis to explain the pathological role of IL17 during ocular *T*. *gondii* infection is that this cytokine might compromise the barrier function of RPE cells, allowing activated immune cells, antigens, and antibodies to cross the BRB, resulting in enhanced inflammation and subsequent tissue damage (Fig 3). Indeed, in vitro studies have shown that IL17 is able to compromise RPE cells monolayers barrier function by disrupting the distribution of tight junctions proteins, like claudins and occludins [83]. Furthermore, IL17 also triggers the recruitment and activation of neutrophils, monocytes, and NK cells to the infection site through the production of IL8, MCP-1, and Granulocyte Colony-Stimulating Factor (G-CSF), as well as stimulates IL6 and NO productions [84–88]. The result of both processes might be a strong amplification of the local retinal inflammatory response in synergy with other mediators, such as IFNγ, TNFα, and IL1, leading to tissue lesions.

These results allow us to outline the immune response to OT (Fig 3). The IL12/IFNγ axis seems to play a central protective role in the eye, similarly to its pivotal and long-known role in systemic toxoplasmosis [50], whereas IL17 has a yet to be defined role in augmenting pathology and interfering in parasite multiplication, probably specific to the eye. There are still numerous gaps in our understanding of the intraocular immune network following *T*. *gondii* infection, such as the role of IL6 and the regulatory roles of T_h_2 cytokines.

## Enhanced virulence: *Toxoplasma* infection in South America

It is important to note that the previous chapters detailed our knowledge on *Toxoplasma* infections in Europe and a great part of North American infections. However, it has become evident that such infections in South American patients are not only more frequent, but also more severe, including the ocular involvement [89]. Indeed, while in Europe and North America, about 2% of infected subjects exhibit OT, the prevalence of this infection in South America seems to be much higher, reaching 6% in Colombia and up to 17.7% of the overall population in Brazil [2,89–91]. A study directly comparing French and Colombian cases showed more macular involvement, larger lesions, more inflammation, and a greatly enhanced ocular parasite load in the Colombian patients [92]. Infections on other continents are yet less studied, but could also reveal striking differences depending on local, more aggressive parasite strains, as suggested by a study, which showed that British residents born in West Africa had a 100-fold higher incidence of OT than people born in Britain [93].

These clinical differences between South American and European patients can be explained by *Toxoplasma* strain differences. Whereas European infections are nearly exclusively attributed to very few, homogenous, and mildly virulent strains, South American strains show a great diversity and often enhanced pathogenicity due to more virulent variants of certain crucial genes, which greatly affect the resulting immune response [94–97]. The above mentioned study [92] compared the expression of cytokines in the aqueous humor of French and Colombian OT patients and found that South American patients expressed pivotal protective factors, such as IFNγ, but also IL17A, in a much weaker fashion, whereas T_h_2 cytokines, like IL13 and, paradoxically, IL6 were up-regulated. This indicates that these highly virulent parasite strains suppress or evade the host control mechanisms, leading to extremely high parasite burdens and, consequently, severe retinal damage, in contrast to the “European-type” milder and probably inflammation-mediated pathology [3]. As there are only very few animal studies using these atypical strains [98], the pathophysiology of these infections is still largely unknown. Interestingly, human genetic disposition, mainly concerning immune response genes, also seems to be an important factor to determine the development of severe disease [99]. The high frequency of pathological cases in South America surely will result in more interesting human studies regarding genetic predisposition.

## Perspectives and opportunities for research

As knowledge regarding OT pathophysiology grows, many questions remain unanswered. Various fundamental aspects of the infectious process leading from *T*. *gondii* oral infection to OT are still unknown. For example, the preferred route for the parasite to invade the retina has not been identified. Evidences show that *T*. *gondii* might cross the BRB either as a free tachyzoite, or inside a “Trojan horse” cell. Furthermore, eBRB and iBRB are very different structures, and it has not been determined which one is preferentially crossed by the parasite, even if some indirect evidences show that the iBRB might be the major route [27]. Once in the retina, the parasites invade cells, but, here again, the main targets remain to be established. Finally, events triggering and mechanisms underlying recurrences remain hypothetical, and further research is critically needed in order to better comprehend this major aspect of OT pathophysiology.

The immune response to OT seems to be widely dependent on IFNγ, which has a pivotal role in the defense against *T*. *gondii*. However, the peculiarities of the eye, its BRBs, and immune environment suggest that other mechanisms might be at stake, as indicated by the peculiar role of IL17. Other possible mechanisms could involve the activation of type I (α and β) or III (λ) IFNs pathways. Indeed, few data exist regarding the role of these IFNs during OT. IFNβ might potentiate the protective effect of IFNγ in a murine model of systemic infection [100]. Nagineni and colleagues also showed that IFNs-α and -β have the ability to inhibit parasite replication in the setting of in vitro RPE cells infections with an RH strain [101]. A similar effect was described in murine macrophages and embryonic human fibroblasts, the immunity-related GTPase M1 protein (IRGM1), which is recruited at the surface of the parasitophorous vacuole, being described as a key effector in this phenomenon [102]. In contrast, another study showed that infection of murine macrophages with atypical virulent strains triggered the expression of IFNβ by these cells and that this production was correlated to parasite death [103]. It might be the consequence of cell stimulation with parasitic debris rather than with live parasites, since exposure of macrophages to heat-inactivated canonical strains caused the same response. Finally, a very recent study showed that type I IFNs are important for the control of parasite proliferation during experimental mouse infection, in particular by promoting the expression of IFNγ by NK cells, and that the toxoplasmic effector *T*. *gondii* inhibitor of STAT transcription (TgIST) allows the parasite to limit the reactivity of cells to type I IFNs by inhibiting the signaling pathway dependent on the STAT1/STAT2 heterodimer [104]. It would be interesting to study this mechanism in OT models.

Regarding type III IFNs, there are currently no data available about their role during toxoplasmosis, ocular or otherwise. However, this cytokine family has been described as of particular importance at natural barriers, such as gastrointestinal epithelium, respiratory epithelium, placenta, or blood–brain barrier (BBB) [105]. Type III IFNs have mainly been studied in the setting of viral infections, but a recent study explored the role IFNλ3 during the infection of the gastrointestinal epithelium with the closely related parasite *Cryptosporidium parvum* [106]. According to this study, these cytokines limit parasite crossing of the epithelium by tightening junctions between cells involved in the barrier as it had already been shown in the BBB in a West Nile virus infection model [107]. A similar phenomenon might be involved in OT pathophysiology, as in vitro RPE cell infection with a virulent *T*. *gondii* strain rapidly increases permeability of the cell layer [108]. Thus, research in this field is required to evaluate the role of such type III IFNs in the regulation of BRB permeability (i.e., through the modulation of intercellular junctions tightness) during toxoplasmic infection and its influence on the course of this infection.

Finally, the identification of IL17 as a potent inflammatory effector during OT, responsible for a strong inflammation and, subsequently, to tissue damage, makes it a potential target for new treatments of OT. Indeed, several compounds, mainly monoclonal antibodies, are already available to inhibit the IL17 pathway (such as ixekizumab, brodalumab, or secukinumab), which are currently under evaluation or in use for treating auto-inflammatory diseases such as psoriasis or ankylosing spondyloarthritis [109–111]. Other therapeutic options could also reside in molecules targeting IL6 or IL23, which are also involved in this inflammatory process. If benefits could be expected from these medications by alleviating inflammation, their antiparasitic effect remains uncertain, and treatment schemes should probably still involve proper antiparasitic drugs. However, immunity-based treatments might be interesting to induce an adapted response to recurrences, both limiting parasite proliferation and tissue lesions. In any case, the implementation of such medications in the treatment of OT needs a more profound understanding of underlying pathophysiological and immunological mechanisms.

## Conclusions

Despite numerous discoveries in the past decade, OT remains a poorly understood disease, which contrasts with its tremendous epidemiological importance. Very basic mechanisms underlying this infection remain unknown, therefore limiting the approaches to develop innovative therapeutics. Indeed, most treatments used nowadays were already used 50 years ago. However, new insights regarding the immune response to OT might provide clues to develop treatment targeting the cytokine pathways responsible for tissue damage subsequently to high inflammation. Thus, further research regarding retina invasion, cyst persistence, recurrences, and inflammatory mechanisms are critically needed in order to develop these new therapeutics, to limit or prevent recurrences or even cure people from chronic infections.

**Key learning points**

- Ocular toxoplasmosis (OT) is an underevaluated clinical problem throughout the world.
- The eye presents a particular immune privileged environment, which considerably influences the local immune response to the infection.
- IL17 has been shown to inhibit parasite control, while enhancing pathology.
- In tropical regions, especially South America, more virulent *Toxoplasma* strains cause more frequent and more severe forms of OT.
- New lines of research studying as yet unexplored aspects of OT pathophysiology, such as the retinal barrier function or reactivation, are necessary to deepen our understanding of this disease and to develop more targeted intervention strategies.

**Top five papers**

Sauer A, Pfaff AW, Villard O, Creuzot-Garcher C, Dalle F, Chiquet C, et al. Interleukin 17A as an effective target for anti-inflammatory and antiparasitic treatment of toxoplasmic uveitis. J Infect Dis. 2012 Oct;206(8):1319–29.
de-la-Torre A, Sauer A, Pfaff AW, Bourcier T, Brunet J, Speeg-Schatz C, et al. Severe South American ocular toxoplasmosis is associated with decreased IFNγ/IL17a and increased IL6/IL13 intraocular levels. PLoS Negl Trop Dis. 2013 Nov;7(11):e2541.
Furtado JM, Bharadwaj AS, Ashander LM, Olivas A, Smith JR. Migration of *Toxoplasma gondii*-infected dendritic cells across human retinal vascular endothelium. Invest Ophthalmol Vis Sci. 2012 Oct 3;53(11):6856–62.
Naranjo-Galvis CA, de-la-Torre A, Mantilla-Muriel LE, et al. Genetic Polymorphisms in Cytokine Genes in Colombian Patients with Ocular Toxoplasmosis. Infect Immun. 2018;86(4):e00597-17. Published 2018 Mar 22. doi:10.1128/IAI.00597-17
Charles E, Joshi S, Ash JD, Fox BA, Farris AD, Bzik DJ, et al. CD4 T-cell suppression by cells from *Toxoplasma gondii*-infected retinas is mediated by surface protein PD-L1. Infect Immun. 2010 Aug;78(8):3484–92.
